# 47例非小细胞肺癌伴神经内分泌分化的临床特点、治疗及预后

**DOI:** 10.3779/j.issn.1009-3419.2019.08.05

**Published:** 2019-08-20

**Authors:** 晓燕 李, 华艳 许, 勋 康, 静 赵, 艺 林, 沙沙 王, 晓晴 刘

**Affiliations:** 1 100070 北京，首都医科大学附属北京天坛医院肿瘤综合治疗病区 Department of Oncologic Comprehensive Therapy, Beijing Tiantan Hospital, Capital Medical University, Beijing 100070, China; 2 100071 北京，解放军总医院第五医学中心肺部肿瘤科 Department of Lung Cancer, The Fifth Medical Center, General Hospital of People's Liberation Army, Beijing 100071, China

**Keywords:** 肺肿瘤, 神经内分泌分化, 驱动基因, 预后, Lung neoplasms, Neuroendocrine differentiation, Driver gene, Prognosis

## Abstract

**背景与目的:**

非小细胞肺癌(non-small cell lung cancer, NSCLC)伴神经内分泌分化(neuroendocrine differentiation, NED)是一个新的病理分类，在临床中并不常见，本文拟探讨NSCLC-NED的临床病理特征、影像学特点、治疗及预后。

**方法:**

收集解放军总医院第五医学中心2009年1月-2017年11月期间收治的47例NSCLC-NED患者的临床资料，总结其人口学资料、影像学特点、病理特征及治疗和预后，分析不同因素与预后之间的相关性。

**结果:**

47例NSCLC-NED患者中，中位年龄61岁(45岁-78岁)，男性38例，女性9例; 37例为低分化癌伴NED，10例为中分化癌伴NED; 2例驱动基因阳性(1例为*EGFR*敏感突变，1例为*ALK*融合)，一线化疗的客观有效率(objective response rate, ORR)为34.5%，中位无进展生存期(progression-free survival, PFS)为4个月; 整体中位总生存期(overall survival, OS)为11个月，OS超过2年者仅2例(4.2%, 2/47)。

**结论:**

NSCLC-NED不同于单纯的NSCLC或肺神经内分泌肿瘤，男性、≤70岁、重度吸烟、肿瘤分化程度较低者较常出现NED，且发病时多为Ⅳ期。该类患者驱动基因阳性比例低于普通腺癌人群，对化疗较不敏感，总生存期偏短，提示预后较差。

## Abstract (en)

**Background and objective:**

Non-small cell lung cancer (NSCLC) with neuroendocrine differentiation (NED) was a new pathologic type and uncommon in clinics. The aim of this study is to observe the relationship between clinical pathologic characteristics, imagination, biological behavior and prognosis in NSCLC-NED.

**Methods:**

The clinical data of 47 patients with NSCLC-NED admitted from January 2009 to November 2017 in the Fifth Medical Center of General Hospital of People's Liberation Army were collected. The demographic data and imaging characteristics were summarized. Pathological features, treatment and prognosis, analysis of the correlation between different factors and prognosis.

**Results:**

Of the 47 patients with NSCLC-NED, the median age was 61 years (45 years-78 years), 38 males and 9 females; 37 were poorly differentiated cancer with NED, and 10 were middle differentiated cancer with NED; 2 cases of driving gene positive (1 case of *EGFR* sensitive mutation, 1 case of *ALK* fusion), objective response rate (ORR) of first-line chemotherapy was 34.5%, and median progression-free survival (PFS) was 4 months; the median overall survival (OS) was 11 months, and only 2 cases (4.2%, 2/47) of OS were over 2 years.

**Conclusion:**

NSCLC-NED is different from simple NSCLC or pulmonary neuroendocrine tumors. Males, ≤70 years old, severely smoking, and patients with lower tumor differentiation often have NED, and most of them are stage Ⅳ. This type of patient-driven gene positive proportion is lower than the general adenocarcinoma population, less sensitive to chemotherapy, and the overall survival is shorter, indicating a poor prognosis.

按照组织病理学分类，非小细胞肺癌(non-small cell lung cancer, NSCLC)占原发性肺癌的80%-85%，主要分为腺癌、鳞癌、腺鳞混合型或大细胞癌^[1]^，其中，1999年世界卫生组织(World Health Organization, WHO)病理分期特别提出一个新的分类：NSCLC伴神经内分泌分化(neuroendocrine differentiation, NED)，指的是：除经典的肺神经内分泌肿瘤(neuroendocrine lung tumor, NELT)外，NSCLC中存在部分肿瘤，虽然不具有光镜下典型的NE细胞形态特点，但相关NED标记物染色阳性并且在电镜下可见细胞内的NED颗粒，表现出同经典NELT相似的特性。WHO将这部分具有NED分化的NSCLC归为Ⅱ类NELT^[2]^。这一病理亚型不同于单纯的NSCLC或肺神经内分泌肿瘤，但在临床实践中对不具有典型光镜下NED形态结构的NSCLC不常规进行相关NED免疫组化染色，导致人们对这一类肿瘤的诊断、治疗及预后认识不足。

本研究通过对47例伴NED的NSCLC病例进行回顾性分析，总结其临床特征、影像学及病理特点，并分析其与治疗和预后的相关性。

## 1 资料与方法

### 1.1 临床资料

通过计算机病案管理系统检索解放军总医院第五医学中心2009年1月-2017年11月临床确诊的NSCLC-NED共47例，采集相关信息进行汇总分析。

### 1.2 纳入与排除标准

结合WHO提出的分类标准^[2]^，确定以下入选标准：(1)均有NSCLC的病理学诊断; (2)均伴有NED; (3)排除肺神经内分泌肿瘤(典型类癌、不典型类癌、小细胞癌、大细胞神经内分泌癌); (4)均有完整的病历资料并接受了生存随访。

## 2 结果

### 2.1 一般资料

47例NSCLC-NED患者中，男38例(38/47, 80.9%)，女9例(9/47, 19.1%); 中位年龄61岁(45岁-78岁)，≤70岁者34例(34/47, 72.3%)，> 70岁者13例(13/47, 27.7%); 37例(37/44, 84.1%)为低分化癌(包括低分化腺癌、低分化鳞癌及低分化癌)伴NED，10例(10/44, 15.9%)为中分化癌(包括中分化腺癌、中分化鳞癌)伴NED; 11例(11/47, 23.4%)为轻度吸烟或不吸烟，36例(36/47, 76.6%)为重度吸烟; 2例(2/47, 4.3%)为驱动基因阳性(*EGFR*突变者1例，*ALK*阳性者1例)，45例为野生型肺腺癌(45/47, 95.7%)，见表 1。

**1:** 47例NSCLC伴NED患者一般资料 General imformation of 47 NSCLC-NED patients

Clinical characteristics	*n*(%)
Gender	
Male	38 (80.9)
Female	9 (19.1)
Age(yr)	
≤70	34 (72.3)
> 70	13 (27.7)
Differentiation grade	
High differentiated with NED	0 (0.0)
Middle differentiated with NED	10 (15.9)
Low differentiated with NED	37 (84.1)
Pathological type	
Adenocarcinoma with NED	30 (63.8)
Squamous carcinoma with NED	11 (23.4)
Undifferentiated with NED	6 (12.8)
Smoking status	
Heavy smoking	36 (76.6)
Light smoking	2 (4.3)
Never smoking	9 (19.1)
Driver mutation	
Positive	2 (4.3)
Negative	45 (95.7)
NSCLC: non-small cell lung cancer; NED: neuroendocrine differentiation.	

### 2.2 肿瘤分期、影像学特点及转移部位数目

31例(66.0%)患者发病时为Ⅳ期，8例(17.0%)为Ⅲb期，6例(12.8%)为Ⅲa期，余2例(4.3%)为Ⅰ期患者。在本研究中，胸部计算机断层扫描(computed tomography, CT)表现为“中心型占位”者有26例(26/47, 55.3%)，表现为“外周型占位”者有21例(21/47, 44.7%)。47例患者中，表现为“纵隔巨大占位”或“肺内巨大占位伴/或不伴坏死、空洞”(螺旋CT中测量直径 > 8 cm)者有18例(18/47, 38.3%)。常见的转移部位为：骨、脑、肺、肾上腺、肝脏、锁骨上区淋巴结，少见的转移部位有：皮下结节、腹膜后淋巴结及胰腺等。31例Ⅳ期患者中，有14例患者出现≥3个转移部位(45.2%)，7例患者出现2个部位转移(22.6%)，10例患者有1个部位转移(32.3%)，见表 2。

**2:** 47例NSCLC伴NED临床特点 Clinical characteristics of 47 NECLC-NED patients

Clinical characteristics	*n* (%)
Clinical stage	
Ⅳ	31 (66.0)
Ⅲ	14 (29.8)
Ⅲa	8 (17.0)
Ⅲb	6 (12.8)
Ⅱ	0 (0.0)
Ⅰ	2 (4.3)
Characteristics of image	
Center type	26 (55.3)
Peripheral type	21 (44.7)
No of metastasis sites	
1	10 (32.3)
2	7 (22.6)
≥3	14 (45.2)
Common metastasis sites	
Brain	13 (27.7)
Adrenal	6 (12.7)
Liver	6 (12.7)
Bone	17 (36.2)
Lung	13 (27.7)
Others^*^	3 (6.4)
^*^: other sites including subcutaneous, retroperitoneal lymph node and pancreas	

### 2.3 病理特征及分子标志物

47例患者均有明确的病理诊断，腺癌伴NED者30例(30/47, 63.8%)，鳞癌伴NED者11例(11/44, 25.0%)，未分化癌伴NED者6例(6/47, 12.8%)，而在这30例腺癌中，低分化癌伴NED者为28例(93.3%)，11例鳞癌患者中，低分化癌伴NED者9例(81.8%)。检测的免疫组化阳性指标(阳性率)有Syn(19/47, 40.4%)、TTF-1(21/47, 44.7%)、CgA(24/47, 51.1%)、CD56(30/47, 63.8%)，CK7(35/47, 74.5%)、Ki-67指数(检测率40.4%，其中指数≥50%者占86.4%)。这47例患者中，仅有1例低分化腺癌检测到*EGFR* 19外显子缺失突变，另1例低分化腺癌检测到*EML4-ALK*基因融合，见表 1。

### 2.4 治疗及预后

可计算总生存期(overall survival, OS)的33例患者中，中位OS为11个月(2.5个月-32.0个月)，OS超过2年者仅2例(2/33, 6.1%)。其中26例Ⅳ期患者(26/33, 78.8%)的中位OS为8.3个月(2.5个月-32个月)，25例低分化癌伴NED患者(25/33, 75.8%)的中位OS为11个月(2.5个月-32个月); 1例患者存在*EGFR* 19外显子缺失突变(未用EGFR-TKI类药物，生存期7个月)，1例患者存在*EML4-ALK*融合基因(仍存活，至今超过2年)。

39例Ⅲb期/Ⅳ期的NSCLC-NED患者的一线治疗方案中，35例采用的是NSCLC的治疗方案(铂二联或TKIs)，4例采用的是小细胞肺癌(small cell lung cancer, SCLC)的治疗方案(VP-16联合DDP或CBP)。一线化疗的有效率为34.5%，中位无进展生存期(progression-free survival, PFS)为4个月。

< 3个转移部位的患者有17例，中位OS为10.5个月，≥3个转移部位的患者有14例，中位OS为8.7个月。初诊时有脑转移的患者10例，中位OS为7.5个月，初诊时无脑转移的患者37例，中位OS为11.8个月。

## 3 讨论

### 3.1 NSCLC-NED的定义

近年来研究发现，10%-20%的NSCLC具有NED特征，与缺乏NED的NSCLC相比，这类肺癌患者对化放疗的敏感性和预后研究尚不深入，对其生物学行为的研究也较少，故WHO并没有将其归入肺癌的神经内分泌肿瘤内，而是在1999年“肺与胸膜肿瘤组织病理学分类标准”中，新增加了NSCLC-NED的概念^[2]^。由于分化的神经内分泌细胞在癌组织成分中不足50%，且以单个细胞或细胞巢的形式分散存在，为癌组织的一种伴随成分，被称为癌伴NED，从而与神经内分泌肿瘤相区别。诊断标准是：采用免疫组化法检测嗜铬蛋白A(chromogranin A, CgA)、突触蛋白(synaptophysin, SYN)或电镜下观察到神经内分泌颗粒，任何一项阳性即表示有NED。目前临床工作中检测NED的主要技术是电镜和免疫组化SP法。

恶性肿瘤细胞向NED方向分化，但它仍具有恶性特征，即在癌病灶中的NED细胞既显示肿瘤主体细胞的结构特点，又显示胞浆内分泌颗粒。正常NED细胞具有类似神经细胞的致密圆形胞体和大量细长而有分支的树状突起，在低分化腺癌中NED细胞呈圆形、卵圆形或不规则形，极性消失，这些细胞的胞核与周围肿瘤一样具有明显的异型性。这些散在的神经内分泌细胞与其所在器官原发癌的类型、分期、分级和预后关系密切。研究^[3]^证实，NED现象广泛存在于前列腺、胃肠道、肺等部位的肿瘤中。

目前对NSCLC-NED的研究越来越多，根据WHO对NSCLC-NED的定义，大多数采用CgA、SYN来检测是否具有NED特点，以1个标记物阳性即判定为NED阳性。随着研究者对神经细胞黏附分子(neural cell adhesion molecule, NCAM)的认识不断深入，越来越多的研究开始联合NCAM分析肿瘤细胞是否具有神经内分泌(neuroendocrine, NE)特征。Sterlacci等^[4]^对405例手术标本中通过检测CgA、SYN和神经细胞黏附分子(NCAM, CD56)的表达来研究NE情况，认为只要有一种表达即可认为存在NED，发现有16.1% NSCLC发生NED。Segawa等^[5]^在130例NSCLC中通过免疫组化检测CgA、SYN、N-CAM和Leu7(CD57)，认为只要有一种表达即可认为存在NED，结果发现有16%具有NED特征，其中腺癌中发生NE的比例为27%。

### 3.2 NSCLC-NED的临床特点

Sundaresan等^[6]^认为腺癌伴NED的发生率高于鳞癌，低分化癌伴NED高于高分化，CgA和SYN一般在腺癌中表达较多，CgA在不同分化程度的肿瘤中表达较接近，而SYN在低分化癌中表达显著，说明肿瘤分化程度越低，越容易形成NED，产生异质性肺癌细胞。

在本次回顾性分析中，选取的47例标本均已病理诊断为伴NED的各种NSCLC，其中腺癌为63.8%，鳞癌为23.4%，可见腺癌中发生NED的比例显著高于鳞癌，与国外研究结果相同。而具有NED特征的NSCLC大多数为低分化，这一点与具有低分化特征的SCLC十分相似。SCLC约95%归因于吸烟，中心型占90%-95%，典型表现为大的中心型原发病灶，伴肺门、纵隔淋巴结广泛转移。而本次分析结果，亦可见肺癌患者中重度吸烟者存在NED的比例非常高，而中心型及纵隔巨大占位较多见，与SCLC的特点十分相似。

### 3.3 NSCLC-NED的发生机制和分子靶标

伴NED的NSCLC具有较高凋亡活性，是其对放、化疗敏感的生物学基础，Apaf-1和Caspase-3高表达发挥了重要作用。刘岩等^[7]^总结了30例NSCLC具有NED的特征，发现其平均凋亡指数及Apaf-1、Caspase-3蛋白表达均显著高于不伴NED者(*P* < 0.05或*P* < 0.01); Apaf-1和Caspase-3表达之间呈正相关(*r*=0.287, *P* < 0.01)，两者与细胞凋亡之间亦呈正相关关系(*r*分别为0.385和0.231，均*P* < 0.05)。腺癌和腺鳞癌组织的Apaf-1蛋白表达阳性率较高(*P* < 0.01)，Caspase-3蛋白表达阳性率在老年患者、中高分化癌和伴淋巴结转移者较高(*P* < 0.05或*P* < 0.01)。

赵坡等^[8]^的研究表明c-kit在神经内分泌癌中的表达：年龄大的阳性率高; 分化低的阳性率高(不典型类癌 > 小细胞癌 > 大细胞癌 > 典型类癌); 分期晚的阳性率高; 男性阳性率高; 肿瘤直径 > 5 cm的阳性率高; 阴性表达者生存期长。以上几项均有统计学差异。

### 3.4 NSCLC-NED的治疗和预后

NSCLC-NED的生物学特征及影像学表现介于NSCLC与SCLC之间，但其对化疗的敏感程度是否介于SCLC和NSCLC之间，国内外研究对此存在争议。

Petrovi等^[9]^应用免疫组化的方法检测116例Ⅳ期和Ⅲ期NSCLC组织中NSE、CgA、Syn的表达水平，发现25%存在NED，存在NED的患者治疗敏感度较高，存在30%以上NE细胞的具有很好的治疗效果，认为NSCLC中发生NED的细胞数量可以决定对紫杉醇联合顺铂方案的治疗效果，成为预后因素。但也有研究显示这些NED的肿瘤具有初次化疗敏感、经再次用药耐药率更高的现象。从而导致临床总生存率与一般NSCLC无明显差异。

目前NSCLC-NED治疗方案比较的研究很少。Derks^[10]^应用两种化疗方案，分别是多西他赛联合顺铂、环磷酰胺联合依托泊苷及卡铂，发现具有NED的患者中位生存期(14.8个月)与非NED的患者(10.7个月)相比具有显著差异，而NSCLC-NED患者对环磷酰胺联合依托泊苷及卡铂方案治疗反应好于多西他赛联合顺铂治疗。

本研究在对一线治疗方案分析后我们也发现似乎应用VP-16联合铂类的效果更好。但由于本次研究纳入的病例数仍偏少，而临床实际治疗时，临床医生对于NSCLC-NED的患者更多选择NSCLC的铂二联方案，所以并未收集到更多方案比较的数据，但现有研究结果也给予了我们新的治疗方案的提示。

综合目前研究结果，在对NSCLC进行亚类分析时可以观察到NED似乎是预后差、生存期短的指标，而在分析NSCLC与NSCLC-NED的预后差异时又有大量证据证明NED与预后无关。在本回顾性分析中，患者的中位OS为11个月。结论不统一可能是由于：(1)NSCLC标本大多由腺癌、鳞癌及典型大细胞癌组成，这些病理类型的选择有所不同，在NSCLC病例中的比例也不同，而根据前述研究结果这三种本身具有NED特点是否具有预后差异本身也不统一; (2)NED判断标准不同，选择的NED的标志物不同，不同标志物对NE的敏感性及特异性不同。

综上所述，本文通过回顾性分析47例NSCLC-NED的患者的临床特征、影像学表现及治疗效果，认为NSCLC伴NED可能是个体肿瘤异质性的一种表现，此类患者较少出现*EGFR*突变及*EML4-ALK*融合基因、男性、≤70岁、重度吸烟、肿瘤分化程度较低者较常出现NED，且发病时多为Ⅳ期，一线治疗选择VP-16联合铂类可取得较好的有效率，对紫杉醇及多西他赛等铂二联方案亦敏感。作为肿瘤异质性的表征之一，NSCLC-NED在临床表现、自然病程、病理改变和治疗反应等方面均有其特点，已成为肺癌研究的新领域。目前仍需大样本的临床分析进一步总结其生物学特性，为针对该类型的个体化治疗提供理论依据。
